# The Board of Health

**Published:** 1882-02

**Authors:** 


### Editorial.

### THE BOARD OF HEALTH.

The management of sanitary interests in great centres of population is among the most important questions now occupying the attention of the profession. Preventive medicine is at last regarded of more vital importance to the public than all the curative and palliative measures adopted to eradicate disease or to mitigate human suffering. We join, therefore, in the expression of an earnest and active interest in this or any other movement, which has for its object the healthfulness of our city and the correction of the many abuses which through selfishness and indifference prevail in this and every other community. It is not our purpose, however, in directing attention to this subject, to discuss the principles of sanitation in their application to our city, but rather to criticize the agencies authorized by the charter through which the community is protected against the introduction of disease from without or the generation of disease within. This agency we call the Board of Health, and whether by definite statutory provision, or through powers granted under the common law, is invested with authority to maintain and guard the sanitary concerns of our municipality.

In the organization of such Boards, some consideration is usually given to the fitness of its individual members, but the framers of our city charter, we presume with a view to economy (certainly not to efficiency), constituted a Board of three ex-officio members, the City Engineer, the Comptroller and the

President of the Common Council. If it had been the object to make a Board absolutely worthless, they could not have been more successful. In making this statement we mean no disrespect to the gentlemen referred to, who, in addition to the onerous duties of the positions to which the people elected them, are, by law, empowered to undertake a work for which they have no aptitude and less inclination; but we wish to say that a board with less knowledge of sanitary matters could not have been formed. What is the result? The executive officer of the Board, the Health Physician, is frequently compelled to assume great responsibilities, in order to meet the exigencies of the hour, trusting to this nondescript Board for endorsement, at a meeting at which he himself may be the only person present.

An effort has been made within a few years to correct this state of things by framing an amendment to the charter in which a new Board would be formed. For reasons which we cannot understand this effort has not been successful. It is time, however, that our city, with its broad area and its rapid increase of population, should be placed under strict sanitary control; and with that end in view, we would encourage the inauguration of a movement to amend the city charter in order that this important object shall be secured. If the question were presented what suggestions have we to offer in the organization of such a Board, we would not hesitate to express our conviction that a Board of Health should be composed of at least five members, of which three should be medical men, to serve without compensation. This Board should serve for at least five years, and should be appointed by the Mayor. To this Board should be entrusted large powers, under wise restrictions, in the menagement of the sanitary concerns of the city, in the correction of abuses, in the abatement of nuisances, in the maintenance of proper safeguards against infectious diseases, and also in enforcement of ordinances enacted to protect the lives of our people. Such a Board would have the merit of both permanency and efficiency, and being placed beyond the political influences which enter into the administration of municipal affairs, would yield positive results in the special field assigned to it. We have not space to enter into the details of a plan which should be early adopted to supplant the system now in operation, but under judicious guidance, we are assured that a movement with this end in view would be received with favor by the public and the profession.

